# Controlled Exposure Study of Air Pollution and T-Wave Alternans in Volunteers without Cardiovascular Disease

**DOI:** 10.1289/ehp.1104171

**Published:** 2012-05-02

**Authors:** Marjan Kusha, Stephane Masse, Talha Farid, Bruce Urch, Frances Silverman, Robert D Brook, Diane R Gold, Iqwal Mangat, Mary Speck, Krishnakumar Nair, Kwaku Poku, Chris Meyer, Murray A Mittleman, Gregory A Wellenius, Kumaraswamy Nanthakumar

**Affiliations:** 1 Division of Cardiology, Toronto General Hospital, Toronto, Ontario, Canada; 2 Gage Occupational & Environmental Health Unit, St. Michael’s Hospital and University of Toronto, Toronto, Ontario, Canada; 3 Institute of Medical Science, University of Toronto, Toronto, Ontario, Canada; 4 Dalla Lana School of Public Health, and; 5 Department of Medicine, University of Toronto, Toronto, Ontario, Canada; 6 Division of Cardiovascular Medicine, University of Michigan, Ann Arbor, Michigan, USA; 7 Channing Laboratory, Department of Medicine, Brigham and Women’s Hospital and Harvard Medical School, Boston, Massachusetts, USA; 8 Division of Cardiology, St. Michael’s Hospital, Toronto, Ontario, Canada; 9 Cardiovascular Epidemiology Research Unit, Harvard Medical School, Harvard School of Public Health, and Beth Israel Deaconess Medical Center, Boston, Massachusetts, USA; 10 Center for Environmental Health and Technology, Brown University, Providence, Rhode Island, USA

**Keywords:** air pollution, arrhythmia, controlled exposure, ozone, particulate matter, T-wave alternans

## Abstract

Background: Epidemiological studies have assessed T-wave alternans (TWA) as a possible mechanism of cardiac arrhythmias related to air pollution in high-risk subjects and have reported associations with increased TWA magnitude.

Objective: In this controlled human exposure study, we assessed the impact of exposure to concentrated ambient particulate matter (CAP) and ozone (O_3_) on T-wave alternans in resting volunteers without preexisting cardiovascular disease.

Methods: Seventeen participants without preexisting cardiovascular disease were randomized to filtered air (FA), CAP (150 μg/m^3^), O_3_ (120 ppb), or combined CAP + O_3_ exposures for 2 hr. Continuous electrocardiograms (ECGs) were recorded at rest and T-wave alternans (TWA) was computed by modified moving average analysis with QRS alignment for the artifact-free intervals of 20 beats along the V2 and V5 leads. Exposure-induced changes in the highest TWA magnitude (TWA_Max_) were estimated for the first and last 5 min of each exposure (TWA_Max__Early and TWA_Max__Late respectively). ΔTWA_Max_ (Late–Early) were compared among exposure groups using analysis of variance.

Results: Mean ± SD values for ΔTWA_Max_ were –2.1 ± 0.4, –2.7 ± 1.1, –1.9 ± 1.5, and –1.2 ± 1.5 in FA, CAP, O_3_, and CAP + O_3_ exposure groups, respectively. No significant differences were observed between pollutant exposures and FA.

Conclusion: In our study of 17 volunteers who had no preexisting cardiovascular disease, we did not observe significant changes in T-wave alternans after 2-hr exposures to CAP, O_3_, or combined CAP + O_3_. This finding, however, does not preclude the possibility of pollution-related effects on TWA at elevated heart rates, such as during exercise, or the possibility of delayed responses.

Numerous studies have linked air pollution to cardiac arrhythmias and sudden cardiovascular mortality (Dockery et al. 2005; Hoek et al. 2001; Ljungman et al. 2008; Peters et al. 2000; Rich et al. 2005; Routledge and Ayres 2005; Santos et al. 2008; Wichmann et al. 1989). Increased risks of cardiovascular events in vulnerable subjects in relation to both short-term and long-term exposure to ozone (O_3_) and ambient particulate matter have been reported (Brook et al. 2004; Ljungman et al. 2008; Rich et al. 2005) but the underlying mechanism is unclear.

Recent studies have linked increased arrhythmogenesis to apparent effects of air pollutants on cardiac repolarization parameters (Hampel et al. 2010; Henneberger et al. 2005; Pekkanen et al. 2002; Samet et al. 2009; Sivagangabalan et al. 2011; Zanobetti et al. 2009). Zanobetti et al. (2009) reported an association between T-wave alternans (TWA) and air pollution in high-risk subjects and an increase in TWA magnitude for patients with coronary artery disease exposed to short-term ambient or indoor black carbon (BC). Henneberger et al. (2005) demonstrated that exposure to increased levels of air pollution was associated with repolarization abnormalities in ischemic heart disease patients that might increase the risk of arrhythmic events. Most of these studies have been observational and only a limited number of controlled exposure studies have examined acute responses to single or multiple polluting agents on repolarization parameters as a possible mechanism responsible for arrhythmogenesis (Samet et al. 2009; Sivagangabalan et al. 2011).

We previously demonstrated that short-term (2-hr) exposure of healthy volunteers to O_3_, concentrated ambient fine particles (CAP), or O_3_ and CAP (Sivagangabalan et al. 2011), increased global spatial dispersion of repolarization, a measure of repolarizaton across the spatially separated myocardial regions. In contrast, TWA provides information on the repolarizing current (specifically calcium cycling) during repolarization of the adjacent myocardial regions with discordant alternans (Narayan 2006). Thus, we tested the hypothesis that exposure to individual pollutants or the combination of both increases TWA in a subset of the same study population.

## Material and Methods

*Study participants.* The study included 17 volunteers (9 females and 8 males) who were 18–38 years of age, who were without any preexisting cardiovascular disease or risk factors, and who were not taking any prescribed medications. Blood pressure (BP), cholesterol, and glucose were obtained, and the following exclusion criteria were used: cholesterol > 240 mg/dL, glucose > 126 mg/dL, hypotension (resting BP < 100/50 mmHg), hypertension (resting BP > 140/90 mmHg), and pregnancy or lactation. BP, heart rate variability (HRV), vascular and hemodynamic measures, blood biomarkers, and spatial dispersion of repolarization responses to these exposures were previously reported for some of these participants (Brook et al. 2009; Sivagangabalan et al. 2011).

The study protocol was approved by the Human Research Ethics Committees of the University of Toronto and St. Michael’s Hospital, and the study was conducted at the Gage Occupational and Environmental Health Unit in Toronto, Ontario, Canada. All participants provided written informed consent.

*Exposure protocol.* All participants were exposed for 2 hr at rest to filtered air (FA) with particles filtered out and with no added O_3_, O_3_ with a target concentration of 120 ppb, CAP with a target mass concentration of 150 μg/m^3^, and a combination of CAP (150 μg/m^3^) and O_3_ (120 ppb).

The order of exposures was randomized, and participants were blinded to the order of exposures. Exposure sessions for the same subject were scheduled 2 weeks apart and at the same time of the day to minimize the influence of circadian variation on the study parameters. At baseline, before entering the exposure chamber, a 12-lead resting electrocardiogram (ECG) was performed using a PC-ECG 1200 (Norav Medical Ltd., Kiryat Bialik, Israel) and BP was measured using an automated oscillometric BP cuff (Oscar-1 or Oscar-2; SunTech Medical Instruments Inc., Raleigh, NC, USA). Participants were acclimatized to the exposure chamber by sitting for 15 min in the chamber before receiving the intended exposure. The 12-lead ECG was recorded continuously during the exposure and monitored on a computer screen outside the chamber by study personnel. BP and heart rate were obtained at baseline, at 30-min intervals during the exposure, and immediately after the exposure.

Details of the facility and the exposures have been described elsewhere (Brook et al. 2002; Sivagangabalan et al. 2011; Urch et al. 2005). In short, for CAP exposures, air was drawn from outside the laboratory and particles > 2.5 μm in aerodynamic diameter (PM_2.5_) were removed and concentrated to predetermined concentrations using a high-flow PM_2.5_ impactor (1,100 L/min). O_3_ was produced by an arc generator and added to the CAP airflow. During FA exposure, particles were removed using a high-efficiency particle arrestor HEPA filter and no O_3_ was added. Pollutant levels were monitored throughout the experiment: O_3_ was monitored using a Dasibi photometric analyzer (model 1008RS; Dasibi Environmental, Glendale, CA, USA) and PM_2.5_ levels were monitored by a tapered element oscillating microbalance (model 1400a; Rupprecht & Patashnick, Albany, NY, USA). The exposure chamber was a modified airtight body plethysmograph, and the pollutant air flow (15–20 L/min) was delivered to the seated subject via a face mask.

*Measures of repolarization parameters.* Changes in cardiac repolarization parameters induced by air pollution can identify patients at risk of cardiac arrhythmia and sudden cardiac death (Henneberger et al. 2005; Liao et al. 2010). Although these parameters may quantify different aspects of repolarization, studies have validated these parameters as factors that contribute to arrhythmic risk. The interval from the peak of the T wave to the end of the T wave (Tp-Te) and QT dispersion (QTd) are two different measures of spatial dispersion of repolarization that have been associated with arrhythmic risk (Antzelevitch 2005; Haarmark et al. 2009; Takenaka et al. 2003; Topilski et al. 2007). TWA, an indication of beat-to-beat alterations in the morphology and amplitude of the T wave, is also associated with a high risk of ventricular arrhythmias (Minkkinen et al. 2009; Nearing and Verrier 2002b; Nieminen et al. 2007; Slawnych et al. 2009; Stein et al. 2008; Verrier et al. 2003).

Multiple approaches for TWA measurement have been described (Martinez and Olmos 2005), but two methods—spectral analysis (Gehi et al. 2005; Kaufman et al. 2006; Minkkinen et al. 2009; Narayan 2006; Nieminen et al. 2007) and the modified moving average (MMA) method (Bloomfield et al. 2002; Martinez and Olmos 2005; Nearing and Verrier 2002b; Stein et al. 2008; Verrier et al. 2003)—have been studied extensively. In healthy people, “alternans” are usually seen in elevated heart rates of about 110 bpm (beats per minute). TWA analysis by spectral analysis requires elevating the heart rate by pacing or exercise, which was not possible for our study. Therefore, we used the MMA method that was described by Nearing and Verrier (2002b). This method allows for TWA measurement during ambulatory ECG monitoring. Thus, we were able to study the effect of air pollutants on Holter tracings in controlled exposure chamber experiments.

*Data acquisition.* ECG data were acquired with PC-ECG 1200 (Norav Medical Ltd.) and processed with the accompanying application software (version 4.5.6; Norav Medical Ltd.). The stored ECG files were later converted to text files and used as the raw electrocardiographic data.

*Analysis of recordings.* ECG data for 17 participants who completed exposures to all pollutants were included in the analysis. Data recordings were coded so that we were blinded to the participant and to the exposure type during the analysis. To evaluate the exposure-induced changes on TWA, data for each exposure were divided into “early” and “late” files for the first and last 5 min of the exposure, respectively. The onset of the QRS complex and the Tp-Te were identified for each file using a custom program developed in MATLAB (version 7.5; Mathworks, Natick, MA, USA).

Preprocessing. For an accurate TWA measurement, it is necessary to preprocess the data and remove artifacts that might interfere with the measure itself. The quality of the data recordings and the automated markers were verified, and the most artifact-free intervals of 20 beats along precordial leads were chosen for the TWA analysis. Noise and baseline wander was also removed to obtain a better quality signal before we analyzed the TWA.

**Figure 1:**
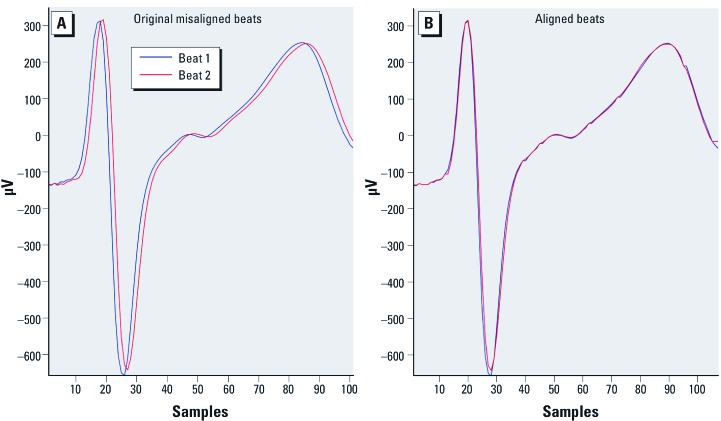
Example of two unaligned beats. (*A*) Repolarization duration is highly heart rate dependent and slight variations in R-R intervals may induce variations in the repolarization duration and in the T-wave misalignment, resulting in inaccurate measurement of TWA with no alignment (44.32 μV). (*B*) QRS alignment using DTW to align them; TWA with QRS alignment (9.56 μV).

QRS alignment. Repolarization duration is highly heart rate dependent and slight variations in R-R intervals may induce variations in the duration of the repolarization and in the misalignment of the T waves (Figure 1A)(Zareba and Moss 1995). TWA refers to the beat-to-beat variations in the morphology and amplitude of the T wave. Thus, the QRS waves must be time aligned (Figure 1B) before comparing the T wave because lack of alignment might artificially result in positive TWA results. The dynamic time warping (DTW) algorithm (Cuesta-Frau et al. 2003) was used for QRS alignment (Cuesta-Frau et al. 2007a, 2007b). DTW is a pattern-matching technique that stretches or compresses the two heartbeats under analysis, in time, in a nonlinear way, to align them.

TWA measurement. TWA was computed offline by study personnel blinded to exposure types from continuous ECG recordings of leads V2 and V5. TWA was measured with MATLAB (version 7.5; Mathworks) based on the MMA method (Nearing and Verrier 2002b), with and without QRS alignment, for the artifact-free intervals of 20 beats using the V2 and V5 leads. Because the MMA method appears to be less heart rate dependent than the spectral TWA method (Cuesta-Frau et al. 2007a, 2007b; Nearing and Verrier 2002a, 2002b), no heart rate criteria were used for the analysis. In brief, 20 beats were divided into odd and even bins, and the morphology of the 10 beats in each bin were averaged to create odd and even moving-average complexes. To avoid the effect of impulsive artifacts, a limiting nonlinearity factor of one-eighth was applied to every new beat. TWA was then computed as the maximum difference in magnitude between the odd-beat and the even-beat average complexes from the J point (the beginning of the ST segment) to the end of the T wave (TWA_Max_). Unless otherwise mentioned, all reported TWA_Max_ values in this article represent the highest TWA value recorded among the analyzed leads (V2 and V5).

*Statistical analysis.* Data were analyzed using the SAS software package (version 9.1; SAS Institute Inc., Cary, NC, USA). We estimated exposure-induced changes in the TWA magnitude based on the difference between the highest TWA value during the first 5 min (TWA_Max__Early) and the last 5 min (TWA_Max__Late) of exposure for each participant: ΔTWA_Max_ = TWA_Max_ _Late – TWA_Max__Early.

Exposure was defined as a four-category variable (O_3_, CAP, O_3_ + CAP, FA). The experimental design was treated as a randomized block with one-way layout of treatment. Analysis of variance (ANOVA) was performed, and we used the contrast statement within ANOVA to compare TWA_Max__Early, TWA_Max__Late, and ΔTWA_Max_ in three exposure groups (CAP, CAP + O_3_, and O_3_) versus FA. TWA_Max__Early, TWA_Max__Late, and ΔTWA_Max_ were treated as outcome variables, and the exposure types and heart rate of the subject were modeled as predictor variables. Heart rate was included in the model to account for any changes in TWA_Max_ and ΔTWA_Max_ secondary to changes in the heart rate. For the ΔTWA_Max_ analysis, TWA_Max__Early was included in the model to adjust for baseline differences in TWA_Max_. Subject effect was also included in the model as a predictor variable. All the values are adjusted for the heart rate and TWA_Max__Early and are expressed as mean ± SE. A *p*-value of < 0.05 was considered statistically significant.

## Results

*Participant demographics.* Data from 17 participants who completed all four exposures were included in the analysis. The demographic characteristics of the participants are shown in Table 1.

**Table 1:** Characteristics of the participants (n = 17).

Characteristic	Females (n = 9)	Males (n = 8)
Age (years)		23.7 ± 4.3		28.1 ± 7.0
Height (cm)		163.6 ± 8.8		177.7 ± 7.8
Weight (kg)		59.7 ± 12.8		75.8 ± 11.7
BMI (kg/m2)		22.3 ± 4.2		24.0 ± 3.3
Systolic BP (mmHg)		115.9 ± 9.9		125.3 ± 8.9
Diastolic BP (mmHg)		71.6 ± 8.8		76.5 ± 7.3
Resting heart rate (bpm)		70.1 ± 9.6		63.8 ± 10.6
BMI, body mass index. Values are mean ± SD.				

*Pollutant characterization during exposure.* In this study, a total of 68 exposures were studied: 17 exposures to CAP, 17 to O_3_, 17 to CAP + O_3_, and 17 to FA (control). The mean CAP mass concentration was 154 ± 54 μg/m^3^, and the mean O_3_ concentration was 109 ± 6 ppb (Table 2). These concentrations are higher than the typical concentrations observed over a 24-hr period in Canada (Campbell et al. 2001; Macfarlane et al. 2000), but such concentrations can occur for 1–2 hr in many North American cities and are commonly encountered in eastern China for longer durations (Fu et al. 2009). No significant differences were observed in mean levels of other gaseous pollutants (carbon monoxide, nitric oxide, nitrogen dioxide, sulfur dioxide) between exposure days (data not shown).

**Table 2:** Exposure-specific pollutant levels.

Exposure	Filtered air	CAP	O_3_	CAP + O_3_
PM2.5 (μg/m3)		2 ± 7		154 ± 54		4 ± 10		147 ± 86
O3 (ppb)		11 ± 8		10 ± 6		109 ± 6		108 ± 5
Values are mean ± SD.								

*Absolute TWA magnitude.* The mean ± SE TWA_Max__Early, which was measured among all participants for the FA exposure (no pollutants), was 10.8 ± 0.7 µV at a mean heart rate of 76 ± 2 bpm. The mean ± SE TWA_Max__Early for O_3_, CAP, and CAP + O_3_ exposure groups was 12.6 ± 1 µV, 11.7 ± 0.9 µV, and 12.3 ± 1.1 µV, respectively (Table 3). All exposure groups were compared with filtered air exposure. There was no significant difference in TWA_Max__Early for CAP versus FA (*p* = 0.2) or for CAP + O_3_ versus FA (*p* = 0.06), but TWA_Max__Early was significantly higher during the first 5 min of O_3_ exposure than during the FA exposure (*p* = 0.03). The mean ± SE TWA_Max__Late for FA, O3, CAP, and CAP + O3 exposures were 9.4 ± 0.6 µV, 9.7 ± 0.6 µV, 9.9 ± 0.7 µV, and 10.6 ± 0.9 µV, respectively (Table 3). There were no significant differences in TWA_Max__Late for CAP versus FA (*p* = 0.6), for CAP + O_3_ versus FA (*p* = 0.09) or O_3_ versus FA (*p* = 0.3).

**Table 3:** Mean heart rate (HR), TWA_Max_, and ∆TWA magnitude results.

Exposure	HR Early (bpm)	HR Late (bpm)	TWA_Max__Early (µv)	TWA_Max__Late (µv)	Mean ∆TWA (µv)
FA		76.2 ± 2.1		78.1 ± 1.7		10.8 ± 0.7		9.4 ± 0.6		–2.1 ± 0.4
O3		74.8 ± 2.1		74.8 ± 2.4		12.6 ± 1.0*		9.7 ± 0.6		–1.9 ± 1.5
CAP		76.6 ± 2.3		76.0 ± 2.1		11.7 ± 0.9		9.9 ± 0.7		–2.7 ± 1.1
CAP+O3		75.8 ± 2.1		77.6 ± 2.0		12.3 ± 1.1		10.6 ± 0.9		–1.2 ± 1.5
Values are mean ± SE. ANOVA model included heart rate, TWAMax_Early. *p < 0.05 compared with FA exposure.										

*Average heart rate at the time of TWA_Max_.* TWA_Max__Early and TWA_Max__Late were regressed against average heart rate during the first and last 5 min of each exposure, respectively. We did not find any significant correlation between TWA_Max__Early and early heart rate (*R*^2^ = 0.01, slope = 0.097, *p* = 0.36), or between TWA_Max__Late and late heart rate (*R*^2^ = 0.001, slope = –0.03, *p* = 0.78) across all four exposures combined.

*ΔTWA_Max_ Analysis (TWA changes over time).* ΔTWA_Max_ was measured with QRS alignment (Table 3). The mean ΔTWA_Max_ was –2.1 ± 0.4, –2.7 ± 1.1, –1.9 ± 1.5, and –1.2 ± 1.5 for the FA, CAP, O_3_, and CAP + O_3_ exposure groups, respectively. There were no statistically significant differences in ΔTWA_Max_ for exposure to any of the air pollutants compared with FA [CAP vs. FA (*p* = 0.7), CAP + O_3_ vs. FA (*p* = 0.6) and O_3_ vs. FA (*p* = 0.8)].

## Discussion

We conducted a controlled exposure study of the specific effects of particulate matter and O_3_ (individually and combined) on TWA in healthy participants and found no evidence of significant impacts. In order to account for intersubject differences at baseline, and because an appropriate duration of exposure is required before the physiological effect can be realized, we believe ΔTWA_Max_ (Late–Early) represents the best measure of the exposure effect. We compared the mean ΔTWA_Max_ for each exposure group versus FA and found no apparent exposure effect upon TWA. However, there was a significant difference in the maximum TWA magnitude during the first 5 min of exposure (TWA_Max__Early) for O_3_ versus FA. Although there is a slight possibility of an immediate effect of O_3_ exposure, we believe that this observation was an artifact of random baseline differences, because the O_3_ concentration was minimal during the first 5 min of the exposure and because it took approximately 20 min for O_3_ to reach the maximum targeted level of 120 ppb in the exposure chamber. Although this was a negative study, in contrast to our previous findings (Sivagangabalan et al. 2011) that showed an increase in global spatial dispersion of repolarization during controlled air pollution exposures, this finding is an important step in systematically understanding the possible mechanism of arrhythmogenesis related to air pollution. Ground-level O_3_ and fine particles are the two primary constituents of urban smog. Even low levels of these pollutants have been associated with arrhythmias in population studies (Macfarlane et al. 2000). The mechanisms of arrhythmogenesis related to air pollution need to be studied for specific exposures, which requires a thorough analysis of the effects of these pollutants on humans. Most epidemiological studies that examine the apparent effects of air pollution on cardiovascular diseases use data obtained from central monitoring sites that lack adequate controls on exposure characterization (Brook et al. 2004; Henneberger et al. 2005; Pekkanen et al. 2002; Routledge and Ayres 2005; Srebot et al. 2009; Zanobetti et al. 2009), which does not allow for a rigorous analysis of individual pollutants or pollutant mixtures. In contrast, we have completed a controlled exposure investigation of the acute estimated effects of air pollution on TWA.

The pollutant concentrations used in this study (150 μg/m^3^ for CAP and 120 ppb for O_3_) are higher than those typically observed over a 24-hr period in Toronto, Canada (Campbell et al. 2001; Macfarlane et al. 2000), but the concentrations of these pollutants can often exceed 150 μg/m^3^ for CAP and 120 ppb for O_3_ for 1–2 hr at a time in many North American cities, and similar levels are commonly encountered for longer durations in eastern China (Fu et al. 2009).

Effects of air pollution on cardiac electrophysiological parameters over time have been previously reported. Cavallari et al. (2008) reported early effects of occupational exposure to PM_2.5_ on HRV 2 hr after exposure and a delayed response 9–13 hr postexposure in a crossover panel study of 36 boilermaker welders. Liao et al. (2010) observed evidence of a significant adverse effect of PM_2.5_ on ventricular repolarization within 3–4 hr of elevated PM_2.5_ in a study of 106 nonsmoking adults with individual measures of PM_2.5_ exposure over a 24-hr period. Sivagangabalan et al. (2011) observed an increase in global spatial dispersion of repolarization in 25 healthy subjects who were exposed to air pollution for 2 hr in a controlled exposure study, and Henneberger et al (Henneberger et al. 2005) observed evidence of an immediate effect of PM_2.5_ air pollution on repolarization parameters (duration, morphology, and variability) among 56 male patients with coronary heart disease. These findings suggest that the 2-hr experimental exposure used in our study was adequate to study the apparent effects of air pollution on TWA.

Associations between air pollution and TWA among susceptible populations have been previously studied (Henneberger et al. 2005; Zanobetti et al. 2009). Associations between daily variations in particulate air pollution and repolarization parameters were assessed in a panel study that included 56 patients with ischemic heart disease in East Germany (Henneberger et al. 2005), and the investigators reported significant changes on repolarization duration, morphology, and variability. In an observational study of 48 patients with coronary artery disease in Boston, Zanobetti et al. (2009) reported that sitting in traffic was associated with an increase in TWA 2 hr later, which suggests an association between air pollution and TWA. However, there are no data regarding the influence of air pollution on TWA among healthy adults. Alterations in TWA that are due to air pollution exposure may only occur in the setting of structural heart disease, which may explain the discordant findings in our current study.

MMA is one method for TWA measurement. We chose the MMA method because it has been shown to be robust to noise, tolerant to nonstationary data (such as motion artifacts and changing heart rates), and independent of phase-shift perturbations, and, unlike the spectral analysis method, it does not require elevated heart rate (Nearing and Verrier 2002a, 2002b).

The results of several experimental studies (Kop et al. 2004; Kovach et al. 2001; Lampert et al. 2000, 2005) suggest that acute mental stress induces cardiac electrical instability and results in TWA. Kop et al.(2004) studied mental stress induced TWA among 23 patients with implantable cardioverter devices (ICDs) and 17 controls; similarly, Lampert et al. (2000, 2005) investigated associations between psychological stress and increased TWA in 33 patients with ICDs. Similar findings were reported in an experimental study of canines by Kovach et al. (2001). Our study participants, who were exposed via a face mask and sitting in an exposure chamber, may have experienced anxiety that could potentially obscure the effects of air pollutants on TWA. To adjust for the effects of anxiety on TWA, we used exposure to FA as a control, during which the participants were seated in the exposure chamber and exposed to FA through a face mask.

The strength of the current study is the controlled exposure levels, which allowed us to study the physiological relationship between specific pollutants and TWA in healthy participants, without the effects of structural abnormalities and treatments such as antiarrhythmic or heart failure medications.

## Limitations

Limitations of the present study need to be acknowledged. In our study of 17 participants with no preexisting cardiovascular disease, we did not detect any consistent evidence of adverse effects of exposure on TWA magnitude (increase in TWA), and it is important to consider that this may have been due to inadequate power. Ambient pollutant exposures before each experimental exposure session might have had an impact on the study results; in future studies this impact could be partly addressed by collecting detailed time–activity diaries or personal monitoring. We measured pollutant effects on TWA in participants seated at rest, simulating a person driving in congested traffic and continually exposed to comparable levels of pollution. However, because TWA is highly heart rate dependent, effects during exercise should also be assessed. Our study was designed to examine acute pollution exposure effects within the span of 2 hr; hence, a further limitation is the inability to assess longer-term pollution exposure effects on TWA. In addition, we did not assess effects on TWA between the first and last 5 min of exposure.

## Conclusion

Under basal (resting) conditions, in 17 participants without preexisting cardiovascular disease, we observed no significant changes in temporal dispersion of repolarization assessed by TWA after 2 hr of exposure to 150 μg/m^3^ CAP, 120 ppb O_3_, or combined CAP + O_3_. This finding, however, does not preclude the possibility of pollution-related effects on TWA at elevated heart rates, such as during exercise, nor does it preclude the possibility of delayed response.
